# Recent Iranian Health System Reform: An Operational Perspective to Improve Health Services Quality

**DOI:** 10.15171/ijhpm.2017.89

**Published:** 2017-08-01

**Authors:** Mahdi Mahdavi, Mahboubeh Parsaeian, Ebrahim Jaafaripooyan, Shahram Ghaffari

**Affiliations:** ^1^ Tehran University of Medical Science, Tehran, Iran.; ^2^ Department of Epidemiology and Biostatistics, School of Public Health, Tehran University of Medical Science, Tehran, Iran.; ^3^ Department of Health Management & Economics, School of Public Health, Tehran University of Medical Science, Tehran, Iran.; ^4^ Social Security Research Institute, Tehran, Iran.

**Keywords:** Health System Reform, Iran, Appointment Planning Systems, Outpatient Scheduling Systems, Service Quality Improvement, Operational management

## Abstract

The operational management of healthcare services is expected to directly touch patient experiences. Iranian Ministry of Health and Medical Education (MoHME) for the first time, as such, has sought to improve the operational management of healthcare delivery within a reform agenda by setting benchmarks for ‘number of visit per hour’ and waiting time in outpatient clinics of about 700 affiliated hospitals. As a new initiative, it has faced with mixed reactions and various doubts have been cast on its successful implementation. This manuscript aims to shed some light on the operational challenges of the initiative and the requirements of its successful implementation.

## Introduction

A key concerning issue commonplace in most developing countries, as highlighted by the World Health Organization (WHO), is not just the small share of their gross domestic product (GDP) pouring into their health sector, but, most importantly, is the poor operational management linked with this spending.^1^ Health operations management (HOM) refers to the steps to plan, control, and improve those activities to enhancing patient health and experiences.^2^ It further strives to reduce the patient waiting time and to minimize the idle time of healthcare resources. In this regard, patient planning and scheduling tools and techniques have been used to facilitate the smooth flow of patients through health settings; to reduce waiting time; to minimize the idle time of healthcare resources, and ultimately to boost the efficient use of expensive personnel and medical equipment.^3^

As a less expensive alternative, the speciality care at outpatient clinics has become an essential structure for service delivery in Iran. However, the quality of services at such clinics has been criticized by professionals, researchers, and laymen due to the long waits, delays, and the short consultation time in those settings.^4,5^ Long waits and delays have been approached by the most recent initiative, Health Sector Transformation Plan (HTP) which strives to improve the operational management of health service delivery in Iran. Instruction 4, out of HTP’s eight policy instructions, seeks to specifically promote the service quality of visits in terms of both waiting and consultation time in clinics and other outpatient settings.^6^ It rightfully addresses the long waits and delays in healthcare settings; however, since launched it has been the subject of various scrutiny^7^ both in regard to the quality of an evidence-basis upon which the instruction is built and to the inadequate considerations to implementation issues. In this manuscript we intend to review the instruction from a HOM perspective and to discuss the implementation issues related to this instruction.

## Historical Background

The *status quo* of patient planning in most public healthcare settings of the country including outpatient clinics has been rather chaotic. Such chaos has always existed with the public healthcare settings given the high load of their patients. It has even exacerbated with the introduction of the HTP which has made healthcare services more accessible and affordable than ever. Despite this, there exist extremely few appointment scheduling systems in operation, thus patients have to usually wait for several hours in the clinics or outpatient departments. In most cases, with very few exceptions, the providers admit their patients on a walk-in basis requiring the patients to attend in the service centres in an ad-hoc and random way. At most, the clinics may occasionally set the day of the visit ahead of time. However, neither does the patient know the time of visit, nor has s/he an estimate of the expected waiting time. In busier clinics, patients may wait for several hours. Consequently, the clinics become extremely overcrowded and medical specialists undergo a tremendous pressure to see a larger number of patients within a specific time range.^8^ This is expected to make patients extremely frustrated and ultimately dissatisfied with the services.

Iranian Ministry of Health and Medical Education (MoHME) has sought to somehow improve the operations management of health services in the country.^9^ Prior to the HTP, in its very first initiative, MoHME had once embarked on total quality management (TQM) tools to institutionalize the culture of quality and to support the providers to become effective in addressing all types of quality deficits such as waiting time.^10,11^ This initiative was of very limited achievements in Iranian healthcare organizations, mainly because of the inadequate attentions paid to its implementation eg, in planning and management commitment to the initiative.^12^

## Health Sector Transformation Plan

Long waiting and short consultation time have never been addressed in a reform agenda as specific as in the HTP. This was designed in line with the fifth 5-year health development national strategies (2011-2016) and mainly with the new President’s manifest in order to achieve the universal health coverage, within which a large amount of money was injected into the health sector early 2014.^9,13^ The HTP is the most recent initiative to improve operational management of health services delivery in Iran, which has started to be effective in about 700 hospitals affiliated with the MoHME in the whole country since May 2014. It was originally initiated due to the highly increased out-of-pocket, around 57% at its highest level in 2013, and catastrophic payments.^14^ As a multi-purpose plan addressing quality, fair financial contribution by all individuals, equity and access, the initiative is of a comprehensive perspective covering a wide range of activities such as health, treatment and medical education. Policy instruction 4 of the HTP specifically seeks to promote the service quality of visits in clinics and other ambulatory care settings.^6^ A part of instruction 4, among other things, sets a benchmark and requires all public hospitals to constrain the number of visits per hour. At the first year of the program, 8 visits per hour of a specialist was planned (except for psychiatrists that should see 6 patients only), which might be reduced in the next year. By this instruction the decision makers in the MoHME were keen to close a yawning gap between consumer expectations and perceptions of service quality and to provide services on a par with international standards and thus to, in a way, improve the responsiveness.^15^ However, after 2 years since the instruction has been introduced, waiting time in the specialized outpatient clinics is still markedly high, fluctuating between 2 to 4 hours,^8,16^ which is way above acceptable benchmarks. This signals that the instruction 4 has not been effectively implemented to reduce waiting time in the outpatient clinics. Despite this, consultation time in the specialized outpatient clinics has slightly increased by the instruction 4.^16^

## Implementation Challenges

From the Implementation Science perspective^17^ the instruction 4 *de facto* seems to be challenging and less likely to be easily implemented. A set of prerequisites are expected to determine the degree to which the instruction is successfully implemented. These include ‘quality of evidence underpinning the instruction,’ ‘capacity of hospitals’ including qualifications of people involved in the implementation, ‘the process of implementation,’ and ‘rules and regulations’ surrounding the instruction.

### Quality of Evidence Underpinning the Instruction

Instruction 4, whilst being an overly effective step towards quality improvement, is fairly debatable with respect to its evidence base supporting it. The strength and quality of evidence, stakeholders’ perception of the relative value of the instruction, the adaptability of the instruction to the local needs and its operational costs are questioned.^17^ Though this instruction was suggested at the right time, it looks fairly simplistic and builds on a rather weak form of evidence. Adaptability to the local conditions is neither a small concern. This instruction only sets a very general, ‘one-size-fits-all benchmark’ for all various specialities and patients, while not every patient is similar in terms of their demographic conditions, nor alike in terms of the severity of their illness. The least expectation is to develop speciality-specific benchmarks. Furthermore, the demand for services usually fluctuates daily, weekly, and seasonally. The teaching tasks of the university hospitals could also add to this complexity. Lastly, the cost of instruction deployment in terms of supply and equipment required is also unclear.

### Hospitals Capacities

The compliance with the instruction is incentivised by payments; however, the implementation guide of the instruction must consider several factors related to the internal capacity of hospitals implementing the instruction. In other words, the use of planning systems as an effective tool for improving patient throughput are closely linked to the operational and implementation issues inherent to planning systems. These issues are availability of computerized scheduling systems, perception of professionals regarding patient planning, facility layout, and context of clinics eg, culture of quality.^18,19^ These issues if not addressed thoroughly may pose serious challenges in moving toward the use of patient planning systems. The current planning systems are by and large computer-based in the mainstream of scheduling systems.

This instruction motivates the hospitals to apply appointment scheduling systems to accomplish the benchmark set. The operational guide of this instruction emphasizes that the hospitals must design appropriate computer-based patient planning and scheduling systems for their clinics and outpatient settings. Currently, few hospitals wherein the HTP are implemented make use of computer-based scheduling systems. Much fewer have even designated a call centre or designed a website to allow patients make an appointment. Considering the first phase of HTP which involved 584 clinics enrolling 9500 medical specialists from different disciplines, the amount of work to be done could be guessed. On the other hand, the use of scheduling in patient planning requires a technical and management capability to understand the demand fluctuation and resource allocation.^20^

A workable scheduling system needs information on the extent and characteristics of demand; that is, on the type of patients and their socio-economic status and its fluctuation. However, the demand information is either non-existent or is dispersed.^21^ Furthermore, such a system should access to information on the available resources in terms of rooms, equipment, specialists, and support staff to meet the demand, whilst there is an uncertainty in the availability of resources, which makes patient planning very difficult. As such, scheduling systems take a great deal of efforts and a long time to develop. Although the knowledge and expertise of scheduling is available, its application in Iranian health settings is rather limited. To the best of our knowledge there are few studies demonstrating the application of scheduling in Iran.^8^

### Implementation Process

The benchmark or any other target for visit time either general or disease-specific can hardly be operationalized by a top-down hierarchical order only.^22^ Implementation needs a plan describing its processes.^12^ It encompasses steps-by-step phases to plan, assimilate and engage people, act and evaluate results.^17^ It is important to engage medical specialists as this, on the one hand, might improve the quality of their clinical services and, on the other, they may perceive patient planning systems as a burden to their work planning, hindering their successful implementation. Overall, the execution can succeed provided that the above prerequisites are in place to support its process.

### Rules and Regulations

External factors such as rules and regulations that surround target organizations of the instruction could influence the implementation process. Since the most target hospitals are public entities they are subject to the state rules and regulations. Spending money for a patient planning system mainly seems a non-core service and thus is constrained by the initiatives intended to cut costs of support services. Therefore, outpatient clinics managers have been prohibited to spend more resources including staff to run and maintain the appointment planning systems.

## Theory and Design of Outpatient Planning Systems

So far it has been debated that instruction 4 has not been effectively implemented due to several hindering factors, particularly a contentious evidence basis and overlooked technical aspects of appointment systems. Nevertheless, no evidence-based standards of acceptable waiting time for outpatient visits either routine primary or speciality care is existing. Even in the United States, two accepted benchmarks; ie, ‘third next available’ (TNA) appointment and ‘office visit cycle time’ have resulted in controversial results.^19^

Because of the argumentative evidences on the performance of benchmarks we fall short of being able to recommend feasible benchmarks for number of visit per hour and patient waiting time for outpatient clinics in Iran. Instead we recommend outpatient clinics to use health operational models to tailor benchmarks to the conditions of a clinic. Operational models, rooted in the HOM, provide a basis for analysis and building solutions for scheduling problems. These tools are built on simulation, queuing theory, value stream mapping, and so forth.^22^ Most Western European countries such as the United Kingdom and the Netherlands have to a large extent resolved long waits and delays using operational models.^18,23,24^ Even in China, Singapore, and India this problem has been partially solved.^25^ We provide a generic operational model that could be used to adjust the appointment schedules to specific conditions of clinics. This model first considers the context of outpatient care and then proposes the design of a replicable appointment system.

### Context of Outpatient Care in Iran

The context of care in Iranian outpatient clinics is perplexing and poses daunting challenges; including the shortage of qualified specialists in remote areas, a high fluctuation of demands and consequently the large variability in the length of appointments and out-of-town patients.^16^ There is also still some inclination mostly with these people because of their experience of the current system, thus they tend not to trust the internet-based and tele-scheduling systems and prefer attending physically in the outpatient settings hours before their due time. Furthermore, the cross-flow of patients between specialties is important given a high prevalence of chronic patients who are in need of different specialties to accomplish a diagnosis or a treatment path.^26^ As demand fluctuates, resource allocation should be also flexible.^20^ Conversely, the resource allocation in Iranian outpatient clinics is currently based on group allocation and fixed rather than to be responsive to the actual demands. As a result, while demands fluctuate daily, weekly, and seasonally, the resources capacities remain constant, which in turn could lead to overcrowding in peak demand and idle resources in low demand. These challenges should be taken into consideration in the design of an appointment system.

### Design of a Replicable Appointment System

A priority step in designing an effective appointment system is to meet some basic preconditions. Iranian health providers have to learn to consider patient and family as a primary factor in designing an appointment system. This will result in an appropriate set of choices, attention to patient preference, and shorter waiting times. The providers also need to develop different solutions to accommodate demand fluctuations caused by out of town patients and cross-flow between specialities. Furthermore, they need to redesign and simplify care models with fewer steps, standard processes, pre-visit tasks by non-physician staff, and internet-based visits.^27^

Some studies prescribe advance booking coupled with daily scheduling to accommodate current demand characteristics.^20^ A daily scheduling improves capacity utilization by letting providers to fit appointment of varying length in a daily schedule, by which providers can take care of deviations from planned clinic time and scheduled appointments. It facilitates servicing walk-in, out-of-town, and cross-flow patients.

Well-designed scheduling system involves three types of decisions: appointment rule, patient classification, and adjust for no-show and walk-in.^28^ An appointment rule is a basic decision which determines the number of patients in each block and length of interval between appointments. Appointment rules determine the amount of reserved capacity for types of demand ie, appointments and for unanticipated urgent calls. Appointment rules also reserve capacity for patients who live out-of-town eg, rural areas who might attend in a clinic in an ad hoc manner. Furthermore, appointment rules can accommodate the cross-flow of patients between specialties.^29^ However, no single appointment rule creates desired outcomes in all clinics, thus appointment rule should be adjusted to specific condition of each clinic. Cayirli et al propose a procedure to tailor rules for the clinics with different environments characterized by the probability of no-show, variability of service time, number of patients per session, and the relative value of doctor’s time to patient’s time. This procedure as claimed by Cayirli et al has proved more effective in minimizing adverse effects of no-shows and walk-ins than traditional appointment rules in literature (for detail of generic operational model for appointment scheduling we refer to Cayirli et al).^28^ By providing adjustment procedures and an open-source decision-support tool this appointment rule can be used to develop appointment schedules for clinics.

## Conclusion

The initiative to tackle waiting time is highly expected to improve the responsiveness of Iranian health system. However, operationalizing this policy to minimize waits with the current capabilities of public healthcare settings seems to be not an easy job. We encourage decision-makers in MoHME to eradicate barriers hindering the successful implementation of the HTP’s instruction 4. We furthermore advocate the use of modern tools and techniques to measure and enhance the patient planning in outpatient clinics. In this vein, we encourage operations management researchers to address patient planning issues and employ the demonstration projects showing the value and use of appointment systems in better quality of services.

## Acknowledgements

We are grateful to Professor Jan Vissers for sharing his insights and comments on the earlier versions of the manuscript.

## Ethical issues

Not applicable.

## Competing interests

Authors declare that they have no competing interests.

## Authors’ contributions

MM had the first idea that was later developed into the current form jointly by EJP. Both MP and SG provided their constructive comments on the final version.

## Authors’ affiliations

^1^Tehran University of Medical Science, Tehran, Iran. ^2^Department of Epidemiology and Biostatistics, School of Public Health, Tehran University of Medical Science, Tehran, Iran. ^3^Department of Health Management & Economics, School of Public Health, Tehran University of Medical Science, Tehran, Iran. ^4^Social Security Research Institute, Tehran, Iran.

## References

[1] Africa SS (2016). Financing for health: where there’s a will. Lancet Glob Health.

[2] Vissers J, Beech R. Health operations management: patient flow logistics in health care. Psychology Press; 2005.

[3] Kujala J, Lillrank P, Kronström V, Peltokorpi A (2006). Time-based management of patient processes. J Heal Organ Manag.

[4] Soleimanpour H, Gholipouri C, Salarilak S (2011). Emergency department patient satisfaction survey in Imam Reza Hospital, Tabriz, Iran. Int J Emerg Med.

[5] Sadjadian A, Kaviani A, Yunesian M, Montazeri A (2004). Patient satisfaction: a descriptive study of a breast care clinic in Iran. Eur J Cancer Care (Engl).

[6] Health Sector Evolution, 2014. Iranian Ministry of Health and Medical Education website. http://www.behdasht.gov.ir/.

[7] Rezaei S, Rahimi Foroushani A, Arab M, Jaafaripooyan E. Effects of the new health reform plan on the performance indicators of Hamedan university hospitals (Persian).

[8] Mohebbifar R, Hasanpoor E, Mohseni M, Sokhanvar M, Khosravizadeh O, Mousavi Isfahani H (2014). Outpatient waiting time in health services and teaching hospitals: a case study in Iran. Glob J Health Sci.

[9] Heshmati B, Joulaei H (2016). Iran’s health-care system in transition. Lancet.

[10] Rad AM (2005). A survey of total quality management in Iran: Barriers to successful implementation in health care organizations. Int J Health Care Qual Assur Inc Leadersh Health Serv.

[11] Mosadeghrad AM (2013). Obstacles to TQM success in health care systems. Int J Health Care Qual Assur.

[12] Mosadeghrad AM (2014). Why TQM does not work in Iranian healthcare organisations. Int J Health Care Qual Assur.

[13] Moradi-Lakeh M, Vosoogh-Moghaddam A (2015). Health sector evolution plan in iran; equity and sustainability concerns. Int J Health Policy Manag.

[14] Kavosi Z, Rashidian A, Pourreza A (2012). Inequality in household catastrophic health care expenditure in a low-income society of Iran. Health Policy Plan.

[15] Musgrove P, Creese A, Preker A, Baeza C, Anell A, Prentice T. Health Systems: Improving Performance. World Health Organization; 2000.

[16] Iranian National Institute of Health Research. Study of Healthcare Utilization in Iran. Tehran; 2014.

[17] Damschroder LJ, Aron DC, Keith RE, Kirsh SR, Alexander JA, Lowery JC (2009). Fostering implementation of health services research findings into practice: a consolidated framework for advancing implementation science. Implement Sci.

[18] Koker A. Healthcare management engineering: What does this fancy term really mean? New York: Springer; 2012. Springer Briefs in Health Care Management and Economics.

[19] Brandenburg L, Gabow P, Steele G. Innovation and Best Practices in Health Care Scheduling. Institute of Medicine; 2015.

[20] Gupta D, Denton B (2008). Appointment scheduling in health care: challenges and opportunities. IIE Trans.

[21] Bahadori MK, Mohammadnejhad SM, Ravangard R, Teymourzadeh E (2014). Using queuing theory and simulation model to optimize hospital pharmacy performance. Iran Red Crescent Med J.

[22] Agrrizi D, Agyemang G, Jaafaripooyan E (2016). Conforming to accreditation in Iranian hospitals. Account Forum.

[23] Mahdavi M, Malmström T, van de Klundert J, Elkhuizen J (2013). Generic operational models in health service operations management: a systematic review. Socioecon Plann Sci.

[24] Zonderland ME, Boucherie RJ, Litvak N, Vleggeert-Lankamp CL (2010). Planning and scheduling of semi-urgent surgeries. Health Care Manag Sci.

[25] Cayirli T, Veral E (2003). Outpatient scheduling in health care: a review of literature. Prod Oper Manag.

[26] Hall R. Handbook of Healthcare System Scheduling. Springer Science+Business Media; 2012.

[27] Ben-Tovim DI, Dougherty ML, O’Connell TJ, McGrath KM (2008). Patient journeys: the process of clinical redesign. Med J Aust.

[28] Cayirli T, Yang KK, Quek SA (2012). A universal appointment rule in the presence of no-shows and walk-ins. Pord Oper Manag.

[29] Cayirli T, Veral E, Rosen H (2006 Feb). Designing appointment scheduling systems for ambulatory care services. Health Care Manag Sci.

